# Coronary artery anomalies and the role of echocardiography in pre-participation screening of athletes: a practical guide

**DOI:** 10.1186/s44156-024-00041-4

**Published:** 2024-02-22

**Authors:** Raghav T. Bhatia, Jan Forster, Melanie Ackrill, Nikhil Chatrath, Gherardo Finocchiaro, Saad Fyyaz, Hamish MacLachlan, Aneil Malhotra, Sarandeep Marwaha, Michael Papadakis, Liam Ring, Sanjay Sharma, David Oxborough, Dhrubo Rakhit

**Affiliations:** 1 Hull University Teaching Hospitals NHS Trust, Kingston-Upon-Hull, UK; 2 Cardiovascular Clinical Academic Group and Cardiology Research Centre, St. George’s, University of London, St. George’s University Hospitals NHS Foundation Trust, London, UK; 3 Leeds Teaching Hospitals NHS Trust, Leeds, UK; 4 University Hospital Dorset, Dorset, UK; 5 Institute of Sport, Manchester Metropolitan University and University of Manchester, Manchester, UK; 6 West Suffolk Hospital NHS Trust, Bury Saint Edmunds, UK; 7 Research Institute for Sports and Exercise Science, Liverpool John Moores University, Liverpool, UK; 8 University Hospital Southampton NHS Foundation Trust, Southampton, UK

**Keywords:** Anomalous, Coronary artery anomalies, Echocardiography, Prevention, Screening, Pre-participation screening, Sports cardiology, Sudden cardiac death

## Abstract

Transthoracic echocardiography is an essential and widely available diagnostic tool for assessing individuals reporting cardiovascular symptoms, monitoring those with established cardiac conditions and for preparticipation screening of athletes. While its use is well-defined in hospital and clinic settings, echocardiography is increasingly being utilised in the community, including in the rapidly expanding sub-speciality of sports cardiology. There is, however, a knowledge and practical gap in the challenging area of the assessment of coronary artery anomalies, which is an important cause of sudden cardiac death, often in asymptomatic athletic individuals. To address this, we present a step-by-step guide to facilitate the recognition and assessment of anomalous coronary arteries using transthoracic echocardiography at the bedside; whilst recognising the importance of performing dedicated cross-sectional imaging, specifically coronary computed tomography (CTCA) where clinically indicated on a case-by-case basis. This guide is intended to be useful for echocardiographers and physicians in their routine clinical practice whilst recognising that echocardiography remains a highly skill-dependent technique that relies on expertise at the bedside.

**Graphical Abstract:**

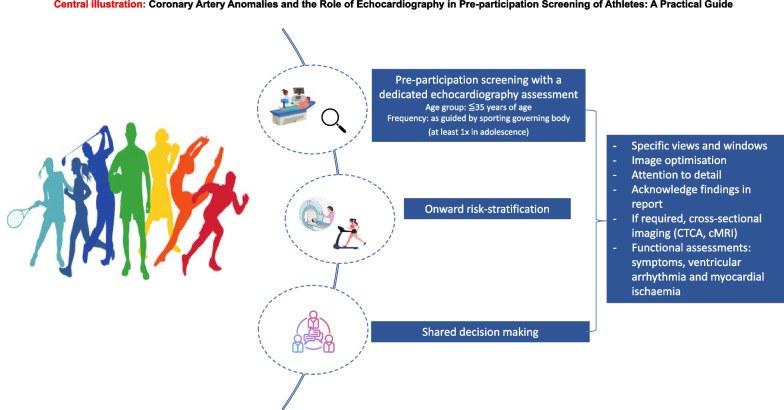

## Introduction

Coronary artery anomalies (CAAs) represent a diverse spectrum of congenital anomalies of the origin of a coronary artery, or the course taken. Clinical presentation and outcomes are highly variable and include incidental findings, to more malignant CAAs associated with sudden cardiac death (SCD). Based on histopathological registries, nuances in study methodology and data primarily relating to young athletic individuals, CAAs have been identified as the underlying aetiology of SCD in up to 17% of cases [1–4]. Furthermore, a potential age and predilection to physical activity may exist, whereby CAAs have been reported to account for 9% of deaths in athletic adolescent individuals, aged between 10 and 19 years, compared to 1% of non-athletic individuals [3].

The premise of cardiac screening in athletes is to detect and manage, often quiescent, cardiac conditions associated with young SCD, which includes a variety of structural and electrical cardiac disorders, including cardiomyopathies, ion channel disorders, congenital disorders and acquired cardiac conditions. Certainly, limitations exist whereby structural abnormalities such as CAAs are not identified on traditional screening modalities, which largely include a clinical consultation followed by a resting 12-lead ECG. Depending on the setting and screening algorithm in place, the addition of a resting transthoracic echocardiogram (TTE) may be the first time a CAA is identified or suspected [5–7].

At present, TTE assessment of the coronary arteries is not a formal requirement in the adult British Society of Echocardiography (BSE) syllabus and is not part of the BSE transthoracic minimum dataset [8]. However, assessment of coronary anomalies is a mandatory skill in the BSE congenital accreditation [9] and as part of cardiac screening of athletes [10].

## Normal coronary anatomy

**Fig. 1:**
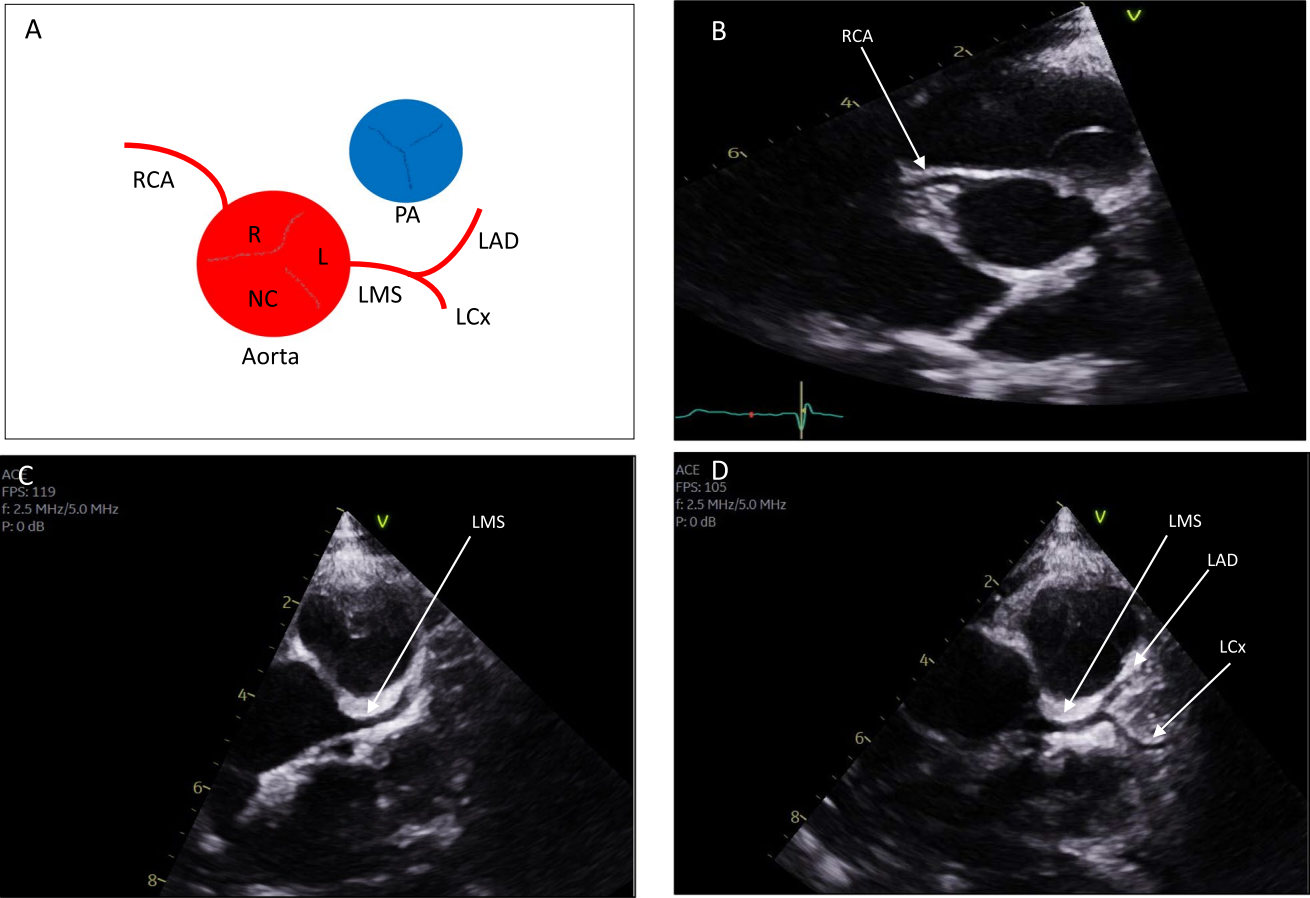
Normal coronary anatomy. **A** Schematic for normal coronary artery origin and course off the aorta in relation to the pulmonary artery; **B**–**D** represent short axis views of the aortic valve; **B** normal origin of the right coronary artery (RCA); **C** normal origin of the left main stem (LMS); **D** normal bifurcation of the LMS, into the left anterior descending (LAD) and circumflex (LCx) arteries

From a TTE perspective, an appreciation of normal epicardial coronary anatomy, in particular the ostia of the coronary arteries and configuration is vital (Fig. 1). The coronary artery ostia originate from the aortic sinuses; left main stem (LMS) from the left sinus of Valsalva and right coronary artery (RCA) from the right sinus of Valsalva. The LMS, typically bifurcates into the left anterior descending (LAD) and circumflex arteries (LCx). A normal variant includes a trifurcation of the LMS to include a ramus intermedius. Each artery may be considered as having a proximal, mid and distal section with branches connected to the coronary microcirculation. Coronary dominance is based on the artery that supplies the posterior descending artery (PDA) and posterolateral branch (PLV) and typically includes a right dominant circulation in 70% of cases.

## Nomenclature of CAAs

Allowing for a plethora of potential anomalies, CAAs can systematically be classified into anomalies of origin, anomalies of course and anomalies of termination.

**Fig. 2:**
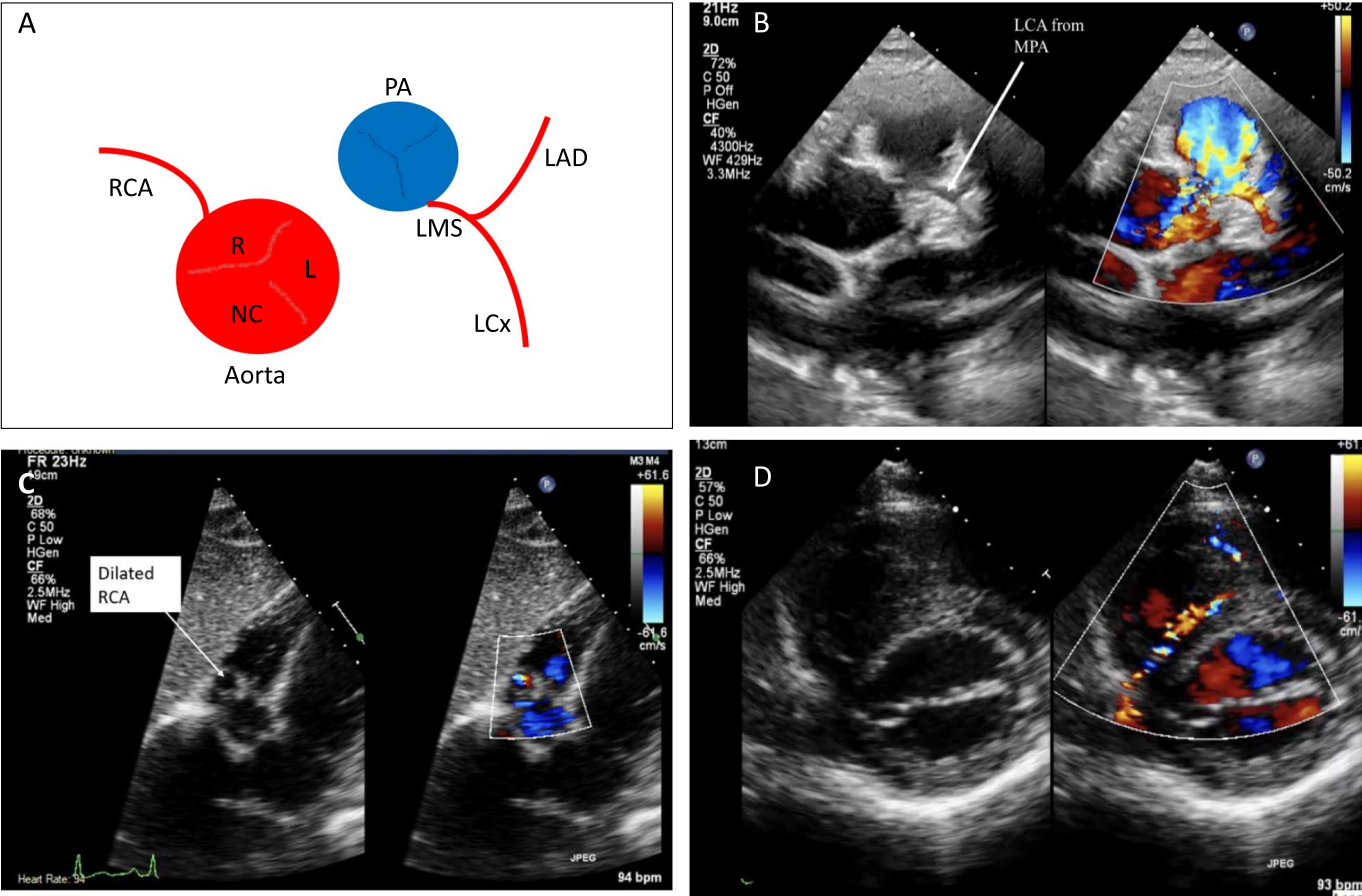
Case of anomalous left coronary artery from the pulmonary artery (ALCAPA). **A** Schematic of anatomical abnormality; **B** LCA from main pulmonary artery (MPA); **C** dilated RCA from subcostal window; **D** anomalous coronary artery flow was visualized along the course of the ventricular septum. TTE findings of ALCAPA include an abnormal left coronary artery arising from the pulmonary artery with retrograde coronary flow (CFM adjusted to low scale frequency). Prominence of the RCA and coronary artery collaterals may also be present. ALCAPA may be misdiagnosed for primary endocardial fibroelastosis due to the increased echogenicity and fibrotic appearance of the papillary muscle associated with this anomaly. It can be best visualised using the high left parasternal short axis window with subtle anterior angulation. Care should be taken as to determine the true origin of the LCA arising from the aorta. Difficulty in defining the LCA, with the presence of a dilated RCA, should further arouse suspicion for ALCAPA

**Fig. 3:**
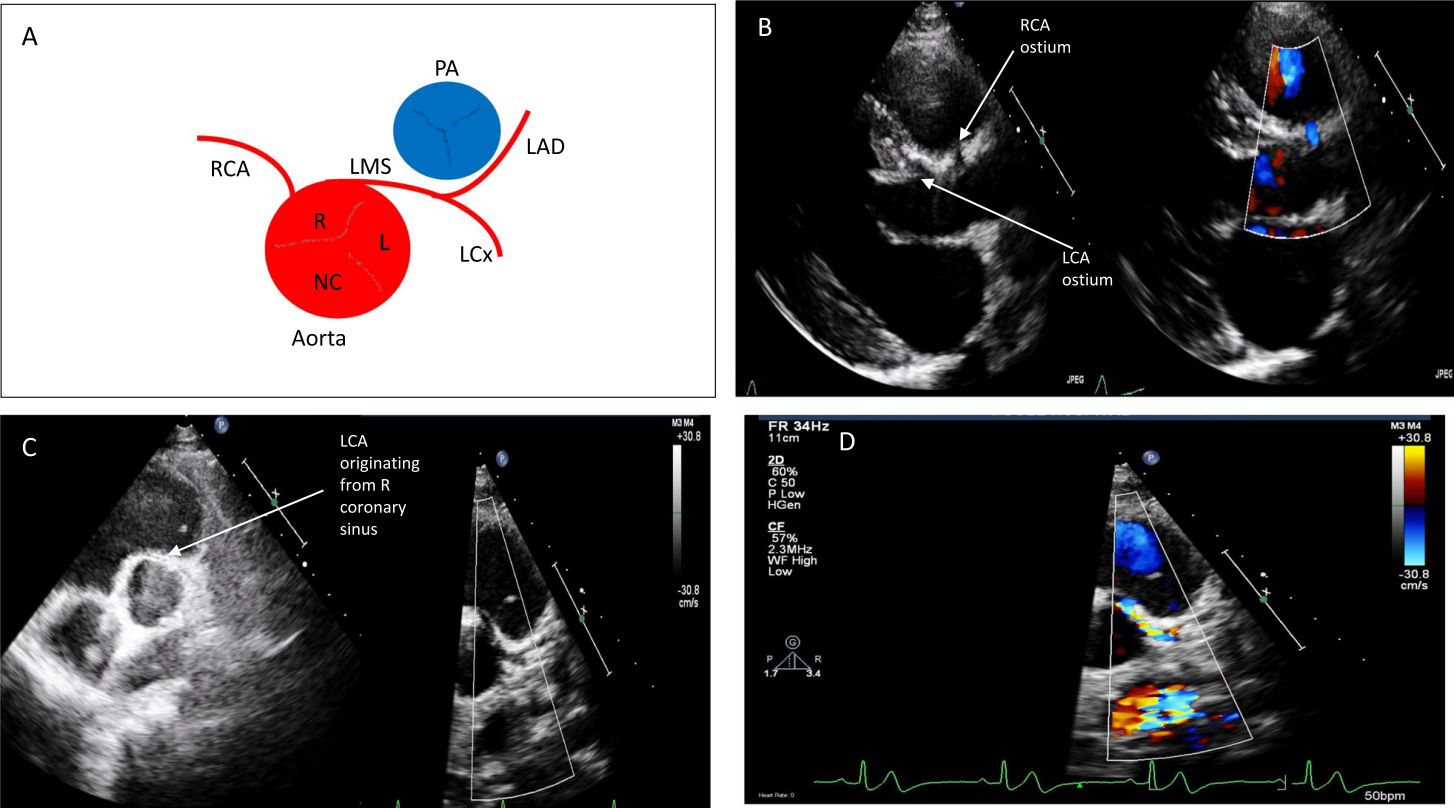
Case of anomalous origin of the left coronary artery from the right coronary sinus (AAOCA) artery with an interarterial course between the great vessels

Anomalies of origin*:* (1) an anomalous coronary artery originating from the pulmonary artery. This includes: the LMS originating from the pulmonary artery (ALCAPA—Fig. 2); RCA from the pulmonary artery (ARCAPA); and LCx from the PA. It is worth acknowledging the rarity of this group (< 0.01%) [11]. (2) An anomalous aortic origin of a coronary artery (AAOCA, Fig. 3). This includes: LMS originating from the right aortic sinus (Fig. 2); RCA originating from the left aortic sinus; LAD originating from the right aortic sinus; LCx originating from the right aortic sinus; and LCx originating from the ostium of the RCA.

Anomalies of course: Anomalies in which the course is posterior to the aorta (retroaortic); anterior to the pulmonary artery (pre pulmonic), interarterial or intramural. Other anomalies include myocardial bridging and coronary artery aneurysms which are beyond the scope of this article.

Anomalies of termination: include coronary arteriovenous fistula and coronary stenosis.

As most congenital malformations may occur in tandem with other structural abnormalities, the same applies to the CAAs. For example, anatomical studies have described abnormal coronary artery ostia and abnormal epicardial courses in individuals with, complete transposition and double outlet right ventricle; persistent truncus arteriosus; and those with tetralogy of Fallot (4–6%) [12, 13]. Appreciating these nuances are important prior to therapeutic strategies. This article will primarily focus on abnormalities of origin and course of coronary arteries.

## Role of echocardiography

TTE serves as the initial cardiac imaging screening modality, in the detection or suspicion of CAAs. In a recent survey, involving over 600 healthcare professionals across 97 countries, 65% of clinicians reported regularly incorporating TTE into their standard screening protocols, citing its ability to identify structural abnormalities, including CAAs, to improve diagnostic yield and prevent SCD in athletes [14].

Despite these real-world findings, there remain important considerations regarding the timing, frequency as well as cost-effectiveness of TTE-based screening. Factors such as the sporting governing body, the age group being screened, and the potential manifestation of latent cardiomyopathic conditions later in life influence these decisions. Taking these factors into account, current recommendations typically support at least one comprehensive echocardiogram during an athlete's career, preferably during adolescence [5, 15, 16].

A recent study by colleagues from Texas has emphasised the pitfalls of a limited two-dimensional screening TTE which inadequately addressed the identification of CAAs. The study primarily focused on detecting structural abnormalities, such as hypertrophic cardiomyopathy, and acknowledged the limitations of existing screening protocols and techniques [17]. Given that CAAs are a common cause of SCD in young athletes, this highlights the need for a more comprehensive evaluation of the coronary arteries using TTE during cardiac screening.

With experience and a dedicated protocol for coronary artery assessment, echocardiography can effectively visualize the origin and proximal course of coronary vessels in over 90% of athletes [15, 18–20]. The assessment can also include the identification of regional wall motion abnormalities and quantification of ventricular function to evaluate myocardial ischemia. As we go on to discuss in this article, the emphasis is on the importance of utilising ad-hoc and non-standardized views, such as sweeping across the aortic valve and ascending aorta, to optimise visualization of the origin and course of coronary arteries (central illustration) [21]. These techniques aim to minimize false-negative results and enhance our understanding of the prevalence and natural history of CAAs. Despite these considerations, limitations exist in assessing higher or lower take-off coronary arteries, mid to distal vessel course, dominance, and intracoronary pathology. In such cases, other non-invasive imaging modalities like computed tomography coronary angiography (CTCA), magnetic resonance imaging (MRI), or invasive coronary angiography may be necessary based on the clinical indication [19, 22].

## CAAs and stigmata of high-risk

Whilst a proportion of athletes are identified with CAAs at a mandatory cardiac screening, individuals reporting CV symptoms, in particular exertional angina and syncope should be thoroughly evaluated for the potential for an underlying CAA. CV symptoms have been reported in up to 37% of individuals, in particular syncope (20%) prior to SCD, where a CAA was attributed as the cause of death [4]. Anomalies in which the course is posterior to the aorta (retroaortic) or anterior to the pulmonary artery (pre pulmonic), are generally not susceptible to compression and are therefore considered benign. Anomalies which precipitate coronary obstruction and malignant ventricular arrhythmia or ischaemia, are usually associated with an interarterial or intramural course. Specifically, an anomalous left coronary artery arising from the right sinus of Valsalva (ALCA) and also anomalous left coronary artery arising from the pulmonary artery (ALCAPA) have been noted in higher proportions in autopsy series from victims of SCD and is thought to confer a greater risk of exercise related sudden death [4, 23, 24]. Additionally, it has been observed that a slit-like orifice or an acute angle take-off of the coronary artery ostia and proximal coronary artery course is associated with a higher prevalence of cardiovascular symptoms, myocardial ischemia, and sudden death [4].

## Principles of onward management

In the absence of randomized control trials and large studies with prospective follow-up, the identification of CAA often poses challenges to clinicians. Based on the appearances of the TTE images, initial management is largely centred around, clearly delineating coronary anatomy with advanced cardiac imaging such as CTCA. Following this, a functional assessment to risk-stratify athletes for SCD ensues. This includes, assessing for the presence of CV symptoms, presence of myocardial ischaemia and exercise induced complex ventricular arrhythmia, by the means of a maximal exercise tolerance test, an exercise stress echocardiogram, or a non-invasive imaging modality such as cardiovascular magnetic resonance imaging (CMR) with perfusion sequences, respectively. In the absence of conventional atherosclerotic risk factors, myocardial enhancement in the late phase following gadolinium in the distribution of a coronary artery territory, signifies evidence of myocardial infarction in the setting of a high-risk CAA [25]. In certain individuals, a more invasive approach with coronary angiography and physiological assessment with intracoronary imaging and pressure-wire studies may be indicated on a case-by-case basis. This is particularly relevant depending on (a) the risk factor profile of individuals, to assess for obstructive or bystander coronary artery disease and perhaps if interventional techniques such as percutaneous coronary intervention are felt to have a role; (b) local expertise and availability of non-invasive imaging modalities; (c) diagnostic confidence in evaluation of the entire course of the coronary arteries—which may be particularly challenging in individuals depending on their resting heart rate and subsequent ECG gating of images with non-invasive modalities [26]. Discussing this in further detail is out of the scope of this article.

A comprehensive understanding of the strengths and limitations of various imaging modalities is imperative. Modern TTE machines exhibit commendable spatial and temporal resolution, with further enhancement of spatial resolution achieved through the integration of coronary computed tomography (CT) and invasive angiography where indicated. While TTE, CT, and cardiac magnetic resonance (CMR) collectively allow for the anatomical evaluation of proximal coronary artery features, such as course and high-risk characteristics including a slit-like ostium, acute angle take-off, and intramural/inter-arterial course, their utility may diminish when assessing distal vessel architecture. Functional assessments predominantly necessitate the evaluation of ischemia and myocardial scar. However, whether physical stress such as exercise in the case of young athletes, which is felt to replicate training and a competitive sport environment compared to pharmacological testing differs in terms of diagnostic accuracy remains an ambiguous area, with a lack of consensus protocols in individuals with anomalous coronary arteries [27]. It is essential to acknowledge that the effectiveness of these evaluations depends on the availability of resources locally, expertise and cost which influences the overall diagnostic accuracy and clinical utility of the imaging modalities.

Following confirmatory and risk-stratification investigations, treatment options are largely based on expert-consensus [22, 28, 29] and take into account an athletes age, physical activity levels, current exercise prescription as well as aspirations [22]. Conservative measures include abstinence from competitive sport or high-intensity training. However, in the presence of (1) CV symptoms, particularly chest tightness during exertion, breathlessness that is disproportionate to the amount of exercise being performed, exertional dizziness or syncope; (2) evidence of exercise related malignant ventricular arrhythmia; (3) evidence of myocardial ischaemia or left-ventricular systolic dysfunction; and/or (4) a high-risk CAA; surgical intervention is advised [19, 22].

## Role of cardiac surgery

Corrective surgery for CAAs is the only potential curative treatment [29]. This decision must involve a congenital cardiologist, and a congenital cardiac surgeon, who has experience of dealing with coronary anomalies. The role of cardiac surgery in individuals including young athletes who are asymptomatic from a CV perspective is less clear, but for high-risk anatomical defects, surgery should be considered to mitigate the risk of SCD [25]. It is of paramount importance to involve the athlete in any decision-making process, as an asymptomatic individual with low-risk anatomy may prefer to follow a conservative approach, with regular surveillance in a dedicated centre with expertise in sports cardiology.

Current guidelines recommend corrective surgery (Level 1C) in patients with ALCAPA, anomalous right coronary artery from the pulmonary artery (ARCAPA) and those with an anomalous aortic origin of the coronary artery with typical anginal symptoms with evidence of stress induced myocardial ischaemia in a matching territory of high-risk anatomy such as an intramural course, orifice anomalies (slit-like, acute-angle take off, orifice > 1 cm above the sinotubular junction) [16, 25].

The surgical techniques are tailored to the specific anatomical characteristics of anomalous coronary arteries [30]. Unroofing techniques are the preferred method of choice for interarterial, or intramural CAAs in young patients. This is performed via an anterior aortotomy, incising the common wall between the aorta and intramural segment of the anomalous coronary artery. Pulmonary artery translocation techniques are performed when the CAA is compressed between the great arteries. Reimplantation or ostial translocation techniques are performed when there is minimal or no intramural component and two separate coronary ostia. Ostioplasty is another surgical technique wherein a neo-ostium is created in the sinus from which the CAA would normally have originated, using autologous pericardium as a patch. It is worth mentioning that bypass grafting outcomes are less satisfactory due to the competitive flow in the anomalous vessel and is generally reserved in cases of atherosclerotic narrowing [30].

Surveillance TTE is recommended post-operatively in high-risk substrates such as ALCAPA to delineate the presence and extent of left ventricular remodelling, and degree of mitral regurgitation [26].

## Safe return-to-play with shared decision-making

Following diagnosis and/or post-surgery, decisions regarding eligibility for competitive sport participation or training at high intensity in individuals of all ages and demographics, particularly in young athletes under the age of 35 years, are challenging and should incorporate a shared decision-making model [22]. This should include the individual, their parents or guardians particularly if under the age of 16-years, sports physicians, cardiologists, surgeons, and club or school coaches. If an individual aspires to partake in high intensity training and competitive sport, with a confirmed CAA that confers a high-risk of SCD, despite medical advice, clear documentation and communication regarding this and involvement of all stakeholders is of paramount importance. Furthermore, in such cases, to mitigate the risk of exercise related SCD, all efforts should be made that an automatic external defibrillator (AED) and personnel that are capable of using it swiftly are available during training sessions and vigorous exercise. After surgical correction, participation in sports may be considered, at 3-months post-surgery, if the athlete is symptom free in the context of a negative exercise stress test [22]. However, this advice depends on the sport and athletes involved in collision sports may be advised to abstain for at least 6-months, to facilitate optimal sternal wound healing. Tailored exercise prescriptions during the convalescence and rehabilitation period, which are supervised by sports physicians and sports cardiologists, that largely involve low to moderate intensity exercise in a graduated manner should be considered on a case-by-case basis with regular ongoing surveillance in a specialist centre.

In recent years, colleagues from North America [31] have formed a CAA registry with a standardised approach to assessment and management. Following assessment of 163 individuals with CAAs of whom 49% were asymptomatic, 50.3% were deemed to have high-risk anomalies. 28% of individuals underwent corrective surgery, all returned back to physical activity with no restrictions post-operatively; and in the overall cohort 82% of individuals were allowed unrestricted sports activities. Such uniform algorithms [31] which emphasise the role of multidisciplinary team members, anatomical and pathophysiological assessment as well as expertise may facilitate optimal risk stratification and long-term prognosis.

## Practical approach for TTE assessment of CAAs

### General aspects

With advances in technology, improved spatial and temporal resolution and harmonic imaging, the assessment of the coronary arteries (CAs) by TTE has become possible. As with all aspects of TTE, optimising patient position and comfort is vitally important. Initial assessment should always begin with the patient positioned in the left lateral decubitus position, with their left arm raised or left hand under their head [8].

### Views

From the parasternal long axis (PLAX), the origin of the right coronary artery (RCA) from the right sinus of Valsalva may be appreciated (Fig. 1, Tables 1 and 2) and this is particularly helpful in identifying high take off RCAs, which arise above the STJ. Furthermore, a discrete circle in the vicinity of the ascending aorta in the PLAX view, should raise suspicion of an interarterial or intramural course between the great vessels. The parasternal short axis view (PSAX) at the level of the aortic valve (AV), is the most useful view to appreciate the origins of the coronary arteries (Tables 1 and 2). As the coronary arteries originate above the plane of the aortic valve, in the PSAX view, patients may need to be rotated further on to their left and the transducer may need to be angled gradually superiorly to appreciate the origins of the coronary arteries above the AV.

**Table 1:** Normal coronary artery assessment

View (modality)	Anatomy	Measurement ± explanatory note	Image
Parasternal long axis (2D)	Ostium of RCA	RCA ostium may be appreciated arising from the right sinus Note: High take-off may also be appreciated as a normal variant	
Parasternal short axis (2D and CFM)	Ostia of RCA and LCA	Visualisation of the origin is usually obtained by tilting more superior to the aortic valve cusps. It may be necessary to come a rib space higher Ensure visualisation of the ostia is in continuity with the aorta RCA ostium may be appreciated at 11–12 o’ clock LCA ostium May be appreciated at approx. 3–4 o’ clock Colour flow imaging may aid visualisation in the coronary artery. The colour scale will need to be reduced to allow flow in the coronary to be appreciated. The direction of the flow in the artery should be noted Colour compare/simultaneous imaging can aid assessment. Use of the cine function allows frame by frame assessment	
Parasternal short axis (2D)	Bifurcation of the LCA	Visualisation of the bifurcation may be obtained by further clockwise rotation. The left anterior descending LAD courses superiorly and the circumflex more inferiorly	
Parasternal short axis (2D)	Trifurcation of the LCA	In some patients it may be possible to demonstrate the trifurcation of the left coronary artery	
Apical 5 chamber view (2D)	Coronary course	The course of the coronary arteries may be appreciated from an aortic outflow view The RCA is seen to course in the A-V groove	
		The LCA is seen to course in the left A-V groove

**Table 2:** Additional examples of coronary artery anomalies

View (modality)	Anatomy	Measurement ± explanatory note	Image
Parasternal short axis (2D)	RCA origin	Prominent RCA origin in comparison to the origin of the LCA	
	LCA origin	Small left coronary with bifurcation not visualised. Circumflex not visualised	
		Retro aortic course of circumflex identified on a modified SAX view	
Modified apical view (2D)		Retro aortic course identified on a modified apical view	
Parasternal short axis (2D)	Dual origin LCA	Dual origin of LAD and CX from the left sinus	
Short axis modified views (2D)	Intramural RCA	RCA arising from left coronary sinus and following an intramural course	
		RCA and LCA arising from a single origin	
		CFM showing flow towards the probe as the RCA takes an intramural course, Flow also seen in the LCA	

In normal circumstances, the RCA normal arises at approximately 11–12 o’ clock position and the left coronary artery (LCA) arises at approximately 3–4 o’ clock position (Table 1). By rotating the probe clockwise, it may be possible to appreciate the bifurcation of the left coronary to demonstrate the LAD and LCx arteries; and counter-clockwise rotation for the RCA. Additional views of the coronary artery courses may be seen in other views. In the apical 4 chamber view, angling anteriorly may demonstrate the course of the LCA circumflex in the left atrioventricular (AV) groove and the course of the RCA in the right AV groove (Table 1).

## Image optimisation

If an adult TTE probe is being utilised, ensure that it is operating it at the highest harmonic frequency. Such an approach may also be utilised with a paediatric probe if the penetration is sufficient for your patient. Many adult TTE systems will have a coronary pre-set which may help you optimise the image. Zooming the image will help you to identify the coronary origins and it is essential to use the cine-freeze function to demonstrate that the artery does in fact connect with the aorta. It is often easier to concentrate on one artery at a time and ensure that it is following a normal course rather than trying to demonstrate both on one frozen image.

## Colour flow mapping

Colour flow mapping (CFM) is useful to visualise flow into the coronary arteries from the aorta. The Nyquist limit needs to be reduced to approximately 30 cm/s; the coronary pre-set on some machines may do this automatically. The cine-freeze function will facilitate the flow more easily and colour comparison or simultaneous functions are particularly helpful. Utilising CFM assists the echocardiographer in excluding pericardial folds or venous coronary structures that may also be appreciated and cause diagnostic dilemmas. Furthermore, CFM may be particularly useful in cases of ALCAPA where the LCA arises from the low-pressure pulmonary artery and retrograde flow may be seen in the coronary artery, creating a coronary steal phenomenon.

## Acknowledging uncertainty

Based on the quality of images, if one or both coronary arteries, and their course has not been systematically visualised in an athlete, it is important to explicitly state this at the time of reporting. This is especially relevant in the setting of CV symptoms whereby clinicians may subsequently undertake additional imaging on a case-by-case basis to clarify coronary anatomy. The ability to accurately identify coronary origins with TTE is largely dependent on the capabilities and experience of the sonographer. The gold standard non-invasive modality to delineate the coronary arteries is CTCA [29].

## Special considerations

Whilst TTE based pre-participation screening is not advocated in those of chronological age < 15 years, in the paediatric setting, it is usual practice to measure the size of the coronary arteries and determine the corresponding Z score for the patients BSA [9]. This is particularly useful if assessing the coronary arteries in the context of Kawasaki’s disease or Paediatric Inflammatory Multisystem Syndrome (PIMS-TS) associated with SARS-CoV-2 infection [32–34].

## TTE tips and tricks for the assessment of CAAs

Whilst the assessment of CAA remains a niche and challenging area, herein we provide 10-take home messages which may support sonographers and physicians looking after athletes (Table 3).

**Table 3:** TTE Tips and Tricks for the assessment of CAAs: 10-take home messages

1	Optimise patient position
2	Allocate sufficient time to identify the ostia of the left and right coronary arteries and potential course taken
3	Standardised and non-standardised views are required
4	Emphasis on the PSAX view, with angulation of the probe above the AV, modified sweeping movements and clockwise and anticlockwise probe rotation
5	Optimise the image (zoom, sector width, and gain)
6	Ensure transducer frequency is optimised for resolution
7	Utilise coronary pre-set if available on machine
8	Utilise colour flow mapping (reduce Nyquist limit to 30 cm/s)
9	Trace the coronary arteries to their origins using the cine/freeze function
10	Acknowledge correct identification of the coronary artery origins in the report, or conversely, highlight any uncertainty in your report if non-diagnostic images of coronary arteries

## Conclusion

Bedside TTE with a dedicated assessment, is pivotal in the initial detection of CAAs in athletes undergoing pre-participation screening. Given that CAAs can lead to sudden cardiac death, particularly among young individuals and athletes, the recognition and tailored risk-stratification with onward management is aimed at conferring significant long-term improvement in mortality and morbidity. Nevertheless, it is essential to acknowledge the limitations of TTE, and clinical assessments should be tailored to the individual's specific circumstances, whilst incorporating the synergistic use of other imaging modalities to augment the diagnostic accuracy.

## Data Availability

The data that support the findings of this manuscript are available from the corresponding author upon reasonable request.
